# Unexpected Durable Treatment-Free Disease Control After Acalabrutinib Cessation: A Case Report

**DOI:** 10.7759/cureus.114638

**Published:** 2026-08-17

**Authors:** Srikaven Sriskandarajah, Ram K Muralidhar

**Affiliations:** 1 Hospital Medicine, North Middlesex University Hospital NHS Trust, London, GBR; 2 Hospital Medicine, Broomfield Hospital, Chelmsford, GBR

**Keywords:** acalabrutinib, bruton tyrosine kinase (btk) inhibitor, cll, targeted therapy, treatment-free remission

## Abstract

We describe a man diagnosed with chronic lymphocytic leukaemia (CLL) at the age of 80 who achieved sustained disease control following discontinuation of acalabrutinib. Initially managed with a watch-and-wait approach, treatment was initiated after evidence of disease progression. Acalabrutinib was commenced but subsequently stopped due to significant adverse effects, including severe anaemia requiring transfusion support. Unexpectedly, serial blood monitoring after cessation demonstrated persistent stability of lymphocyte counts without further CLL-directed therapy. To our knowledge, this represents a rare example of durable treatment‑free disease control following acalabrutinib discontinuation, highlighting the need for further research into predictors of sustained remission after targeted therapy withdrawal.

## Introduction

Chronic lymphocytic leukaemia (CLL) is the most frequent type of leukaemia in adults [1]. It is defined as a lymphoproliferative disorder marked by monoclonal B-cell proliferation. Although the initial mechanism of what triggers CLL is not fully understood, associated risk factors include older age, tobacco use, Caucasian descent, and family history [2].

The main clinical parameter for diagnosing CLL is a raised lymphocyte count of ≥5 × 109/L. In the majority of cases, this is often found coincidentally on routine blood tests [3]. However, cancerous cells may accumulate within the bone marrow. This may impair normal haematopoiesis, causing anaemia, neutropenia, and thrombocytopenia, resulting in symptoms such as fatigue, recurrent infections, and an increased risk of bleeding.

Along with a full blood count, other investigations are used to confirm CLL. Flow cytometry identifies characteristic immunophenotyping of abnormal B lymphocytes, confirming their clonality. Peripheral blood smears give a morphological view of diseased lymphocytes.

In Europe, clinical staging is commonly determined using the Binet classification system, which categorises disease severity based on the number of involved lymphoid areas and the presence of cytopenias. Stage A describes involvement of fewer than three lymphoid regions, Stage B involves three or more, and Stage C is defined by the presence of anaemia and/or thrombocytopenia regardless of lymphoid involvement.

The European Society for Medical Oncology broadly categorises management of CLL into early-stage and advanced-stage disease. Early-stage disease generally refers to patients grouped in Binet A or Binet B, who are typically managed with a watch-and-wait approach. In contrast, management of those grouped in Binet C is individualised and may include chemotherapy or targeted therapy [4].

As CLL remains incurable, treatment focuses on suppressing disease progression and improving quality of life. Historically, chemotherapy regimens were the mainstay of treatment; however, targeted therapies have now become an important component of CLL management [1]. In 2021, NICE recommended acalabrutinib as an option for adults with CLL in specified clinical circumstances, including previously untreated patients with a 17p deletion or tumour protein p53 (TP53) mutation, as well as previously treated patients [5]. Acalabrutinib is a second-generation Bruton’s tyrosine kinase (BTK) inhibitor that suppresses the proliferation and survival of malignant B cells by inhibiting BTK, a key component of B-cell receptor signalling. Generally, it is administered as continuous therapy to suppress disease progression; hence, disease control following treatment discontinuation is not routinely expected. The following case describes a patient who maintained disease control following suspension of acalabrutinib, highlighting the potential for durable treatment-free disease control in selected patients.

## Case presentation

**Figure 1:**
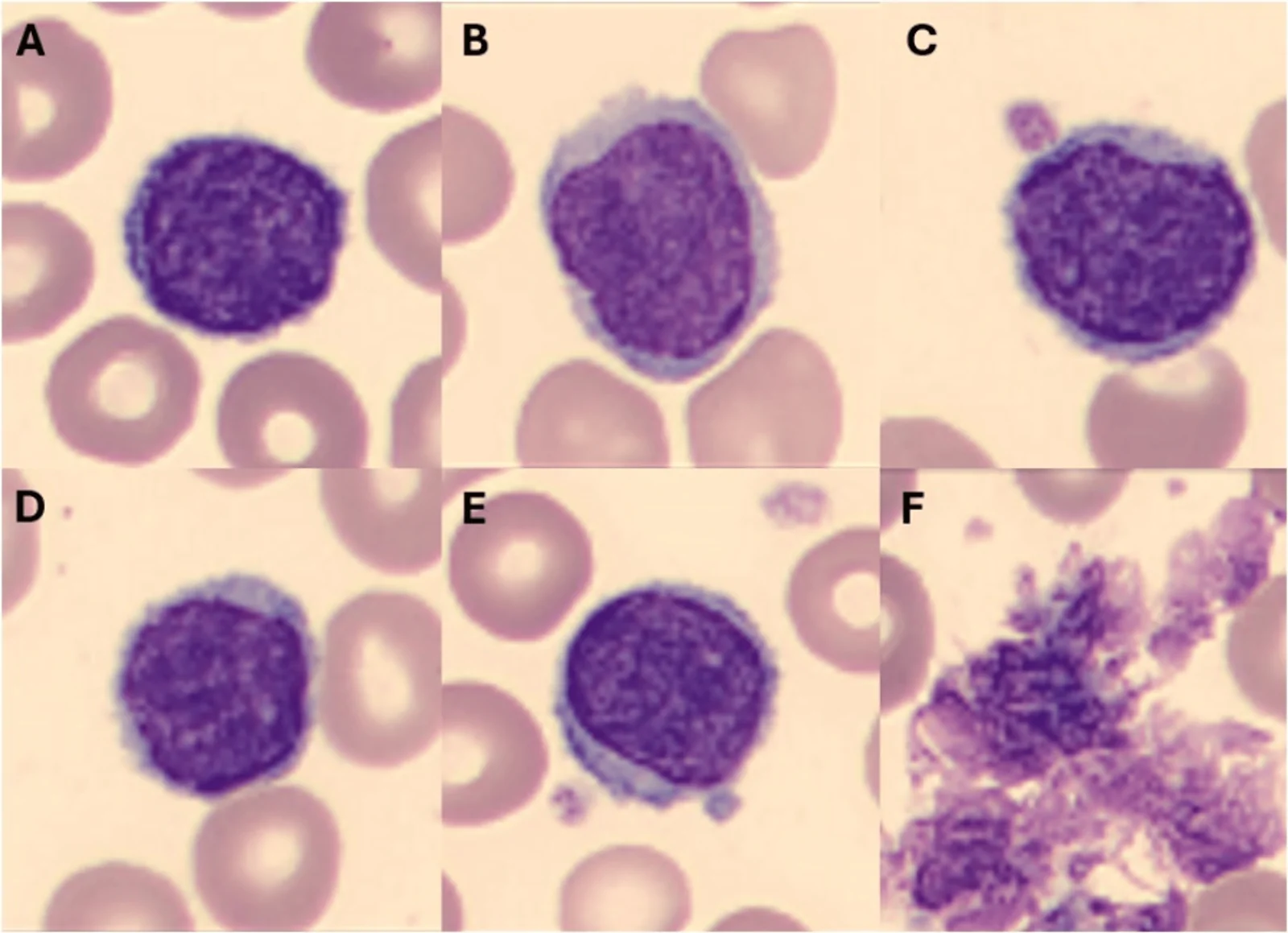
Peripheral blood film at diagnosis. Panels A-E show representative mature lymphocytes with condensed chromatin and scant cytoplasm. Panel F demonstrates a smudge cell, reflecting lymphocyte fragility during smear preparation.

In 2019, an 80-year-old gentleman with no significant past medical history presented with a pruritic red rash covering the posterior torso. He reported no other symptoms, including B-symptoms, and there were no further findings on examination. Initial investigations within primary care revealed a lymphocyte count of 78.2x10^9^ /L (reference range 1.0 - 4.8 x 10⁹/L). Consequently, a two-week wait referral to haematology was arranged. Further haematological investigations (Table 1 and Figure 1) confirmed a diagnosis of CLL.

**Table 1:** Cytogenetic and immunophenotypic findings. A TP53 mutation represents an adverse prognostic marker and is associated with reduced responsiveness to chemoimmunotherapy [6]. An isolated 13q14 deletion is a common cytogenetic abnormality in CLL. For reference, flow cytometry showed CD19+, CD5+, and CD23+, with lambda light-chain restriction. CLL: Chronic lymphocytic leukaemia

Investigation	Result
TP53 mutation analysis	Pathogenic TP53 exon seven mutation detected
Fluorescence in situ hybridisation (FISH)	Biallelic deletion of 13q14 (D13S319) detected
Flow cytometry	Findings consistent with CLL
Ataxia telangiectasia mutated (ATM) deletion	Not detected
Trisomy 12	Not detected
17p deletion	Not detected

As the patient was asymptomatic, a watch-and-wait approach was implemented, with regular clinical reviews and blood count monitoring. Further follow-ups were interrupted during the COVID-19 pandemic, although reasons could not be determined from records. This was re-established in November 2021, when blood tests revealed a persistent anaemia, likely secondary to CLL, as well as a rising lymphocyte count. Following discussion of treatment options, acalabrutinib was decided, commencing in February 2022, when the patient was 83 years old.

**Figure 2:**
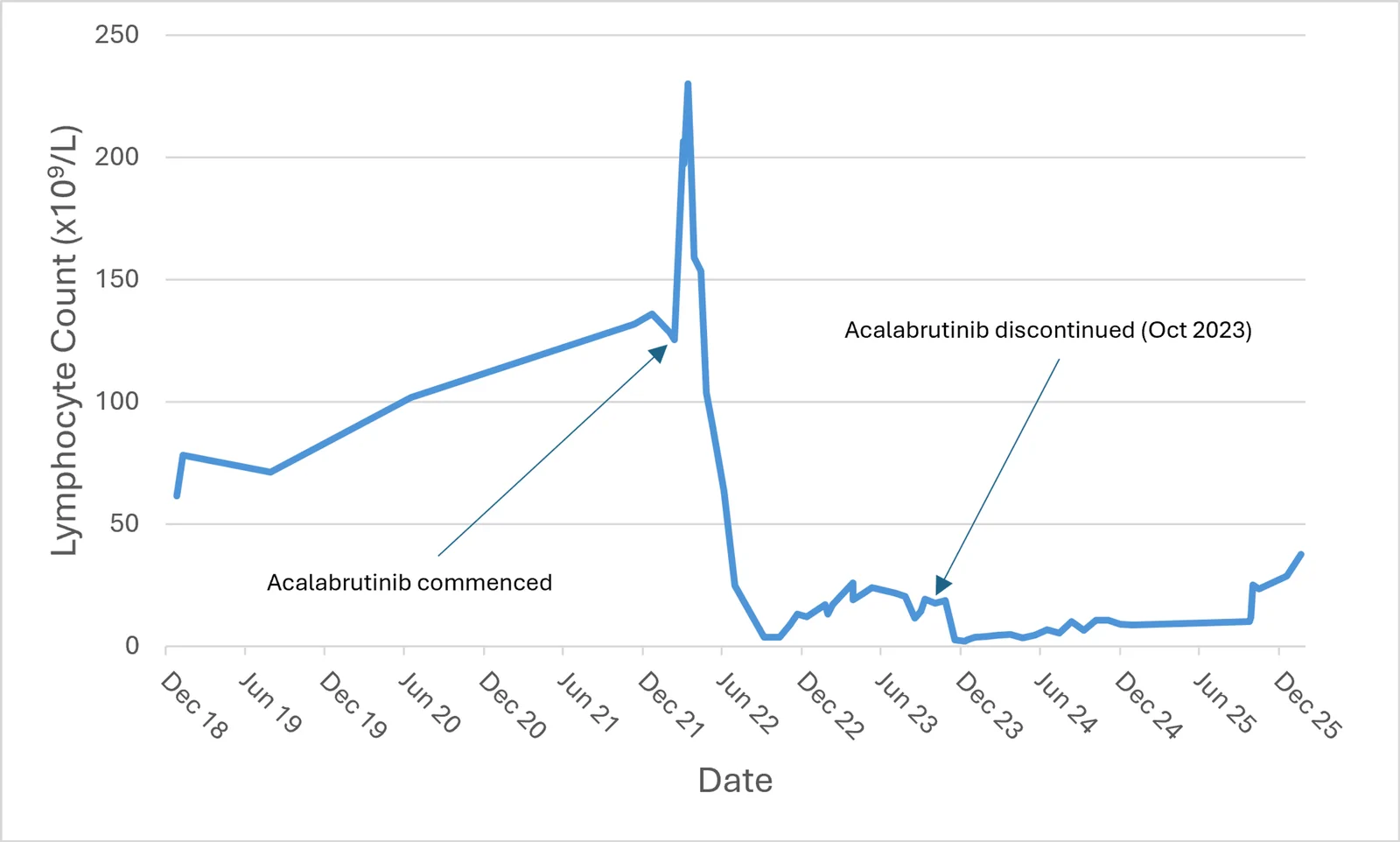
Lymphocyte count progression from 2018 to early 2026.

Initially, an increase in lymphocyte count was observed, likely reflecting redistribution lymphocytosis, a well-recognised pharmacodynamic effect of BTK inhibitors. Whereby malignant lymphocytes are expelled from lymphoid tissue into peripheral blood [7]. Subsequent monitoring demonstrated a progressive reduction in lymphocyte count to 3.4x10⁹/L by December 2022. The overall trend in lymphocyte count following treatment initiation is illustrated in Figure 2.

**Figure 3:**
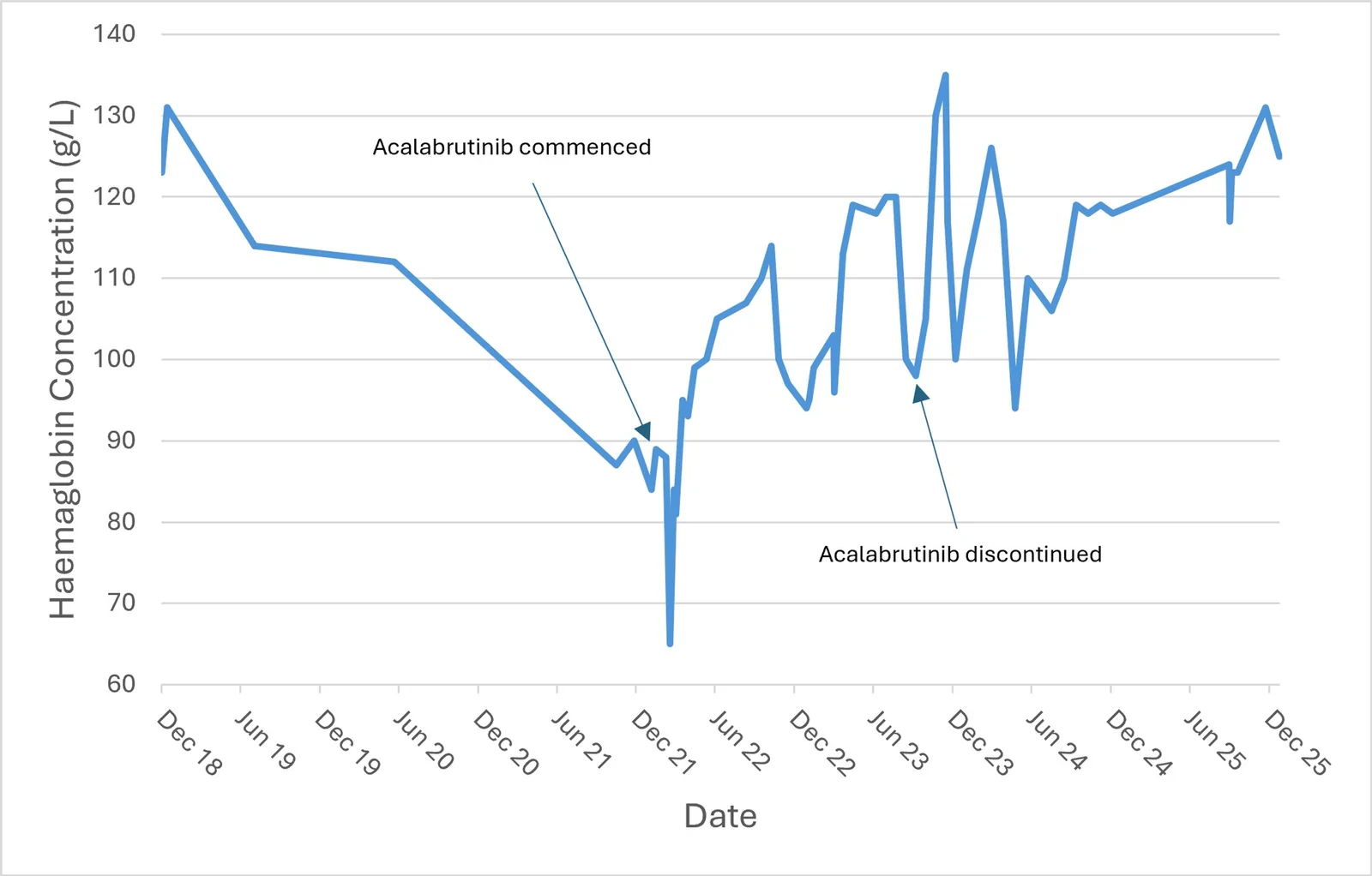
Haemoglobin concentration over time from 2018 to early 2026.

Despite a favourable haematological response, the treatment course was complicated by adverse effects, including persistent anaemia. Although anaemia is a recognised side effect of acalabrutinib, it may also result from underlying bone marrow infiltration by CLL. Shortly after commencing acalabrutinib, the patient’s haemoglobin concentration declined to 65g/L (reference range 130-180g/L), necessitating an emergency blood transfusion, followed by fortnightly transfusions to maintain adequate levels. Overall haemoglobin concentration fluctuated considerably over the course of treatment and further follow-up, as illustrated in Figure 3.

In addition to persistent anaemia, the patient also developed myalgia and bruising during treatment. As therapy progressed, these symptoms worsened, significantly affecting his quality of life. Consequently, a clinical decision was made to temporarily suspend acalabrutinib for one month to allow symptom resolution.

During this period, the patient's symptoms improved and plans were made to recommence acalabrutinib. However, routine blood tests performed prior to re-initiation demonstrated a normal lymphocyte count. Continued monitoring revealed lymphocyte counts ranging from 2.4 to 10.7 × 10⁹/L. Although occasional elevations were observed, lymphocyte counts remained substantially lower than those recorded prior to treatment initiation. Consequently, acalabrutinib was not recommenced, and at the time of writing, the patient, now 87 years old, has remained off acalabrutinib with sustained disease control. He continues to undergo regular haematological follow-up.

## Discussion

At the time of writing, acalabrutinib discontinuation has been in place for approximately 30 months. Haemoglobin levels have returned to normal ranges, with no further transfusions needed after 2023. Although the lymphocyte count has gradually risen during subsequent follow-up, as demonstrated in Table 2, the patient remains asymptomatic. Thus, no further treatment has been recommenced, though monitoring will continue for the foreseeable future, if treatment reinitiation is needed.

**Table 2:** Most recent lymphocyte count and haemoglobin levels following discontinuation of acalabrutinib.

Date	Lymphocyte Count (×10⁹/L)		Haemoglobin Concentration (g/L)
Reference Ranges	1.0-4.8	130-180	
Mar 2025	14.0	102	
Oct 2025	28.6	123	
Jan 2026	37.6	125	

The superior efficacy of BTK inhibitors compared with chemoimmunotherapy regimens reflects the growing understanding of B-cell receptor signalling and pathogenesis of CLL. This has enabled the development of targeted therapies, improving outcomes across multiple patient subgroups. In treatment-naïve CLL, acalabrutinib has demonstrated significantly improved progression-free survival compared with chemoimmunotherapy [8]. This benefit extends to patients with TP53 abnormalities, a subgroup, including our patient, historically known to respond poorly to traditional therapy, in whom BTK inhibitor-based therapy has produced substantially better long-term outcomes [9].

The safety profile observed in our patient is broadly consistent with other published data. In the ELEVATE-TN trial, anaemia of any grade occurred in 14.0% of patients receiving first-line acalabrutinib monotherapy, with grade ≥3 anaemia, generally requiring transfusion, occurring in 6.7% [8]. Although severe anaemia is uncommon in this trial population, these findings suggest that significant haematological toxicity is a recognised adverse effect of acalabrutinib, supporting that our patient's presentation is unusual but not unprecedented.

In the literature, prolonged disease control following permanent cessation of acalabrutinib is not commonly described. The increasing use of targeted therapies has raised an important unanswered question: whether selected patients can safely discontinue BTK inhibition while maintaining durable disease control. Emerging data are beginning to address this question; for example, one phase 1b/2 trial combining ibrutinib with obinutuzumab reported sustained remission in the majority of patients who discontinued treatment after achieving a complete response [10]. Further ongoing clinical trials aim to address this question and may identify biological factors that predict which patients are most likely to sustain remission following treatment cessation as well as those at greater risk of developing significant adverse effects, facilitating a more personalised approach to CLL management.

## Conclusions

This case outlines the events of an elderly gentleman with a new diagnosis of CLL, achieving treatment-free disease control following permanent discontinuation of acalabrutinib. These events contrast the conventional expectation that BTK inhibitors require continuous administration to maintain disease suppression. However, subsequent follow-up is currently demonstrating a rising lymphocyte count, which could suggest relapse, although the significance and trajectory of this rise remain uncertain. As targeted therapies continue to re-evaluate management of CLL, this case highlights the importance of recognising significant adverse events whilst also suggesting that a small subset of patients may maintain disease control, despite treatment discontinuation.

## References

[1] Hallek M (2025). Chronic lymphocytic leukemia: 2025 update on the epidemiology, pathogenesis, diagnosis, and therapy. Am J Hematol.

[2] Brown JR (2024). Clinical risks for chronic lymphocytic leukemia. J Natl Compr Canc Netw.

[3] Mukkamalla SK, Taneja A, Malipeddi D, Master SR (2026). Chronic lymphocytic leukemia. StatPearls [Internet].

[4] Hallek M (2019). Chronic lymphocytic leukemia: 2020 update on diagnosis, risk stratification and treatment. Am J Hematol.

[5] (2026). Acalabrutinib for treating chronic lymphocytic leukaemia. https://www.nice.org.uk/guidance/ta689.

[6] Campo E, Cymbalista F, Ghia P (2018). TP53 aberrations in chronic lymphocytic leukemia: an overview of the clinical implications of improved diagnostics. Haematologica.

[7] Barrientos JC, Burger JA, Byrd JC (2019). Characterizing the kinetics of lymphocytosis in patients with chronic lymphocytic leukemia treated with single-agent ibrutinib. Leuk Lymphoma.

[8] Sharman JP, Egyed M, Jurczak W (2020). Acalabrutinib with or without obinutuzumab versus chlorambucil and obinutuzmab for treatment-naive chronic lymphocytic leukaemia (ELEVATE TN): a randomised, controlled, phase 3 trial. Lancet.

[9] Allan JN, Shanafelt T, Wiestner A (2022). Long-term efficacy of first-line ibrutinib treatment for chronic lymphocytic leukaemia in patients with TP53 aberrations: a pooled analysis from four clinical trials. Br J Haematol.

[10] Castro JE, Lengerke-Diaz PA, Velez Lujan J (2022). Ibrutinib plus obinutuzumab as frontline therapy for chronic lymphocytic leukemia is associated with a lower rate of infusion-related reactions and with sustained remissions after ibrutinib discontinuation: a single-arm, open-label, phase 1b/2 clinical trial NCT0231576. Adv Hematol.

